# Quality of life of people living with HIV, preliminary results from IANUA (Investigation on Antiretroviral Therapy) study

**DOI:** 10.7448/IAS.17.4.19581

**Published:** 2014-11-02

**Authors:** Alberto Venturini, Barbara Giannini, Marcello Montefiori, Antonio Di Biagio, Giovanni Mazzarello, Giovanni Cenderello, Mauro Giacomini, Caterina Merlano, Patrizia Orcamo, Maurizio Setti, Claudio Viscoli, Giovanni Cassola

**Affiliations:** 1 EO Ospedali Galliera, SC Infectious Diseases, Genova, Italy; 2 Università di Genova, Dipartimento di Informatica, Bioingeneria, Robotica, Genova, Italy; 3 Università di Genova, Dipartimento di Economia, Genova, Italy; 4 IRCCS AOU San Martino-IST, Clinica Malattie Infettive, Università di Genova, Genova, Italy; 5 Università degli Studi di Genova, Dipartimento di Informatica, Bioingeneria, Robotica, Genova, Italy; 6 Regione Liguria, Agenzia Regionale Sanitaria, Genova, Italy; 7 IRCCS AOU San Martino-IST, Clinica di Medicina Interna ad Orientamento Immuno, Genova, Italy

## Abstract

**Introduction:**

The introduction of combined antiretroviral treatment (cART) has reduced HIV-associated morbidity and mortality, and changed the patients’ perspective of life. As a result, Health Related Quality of Life (HRQOL) has become a crucial clinical issue.

**Objective:**

Assessment of HRQOL in a sample of Italian patients from IANUA study. Investigate correlation between CD4 cell counts, viral load and changes in HRQOL.

**Materials and Methods:**

EQ-5D-3L self-reported questionnaire has been used in the evaluation of HRQOL. It assesses five dimensions: “mobility,” “self care,” “usual activities,” “pain/discomfort” and “anxiety/depression.” Each dimension has three levels: no problems, some problems and extreme problems. In addition, it includes a Visual Analogue Scale (VAS) where one's own health “today” is rated from 0 “worst imaginable health” to 100 “best imaginable health.” The respondents provide information on marital status, education, employment/unemployment, other treatments used in addition to HAART (1,2,3,4,5 or more) and number of hospitalizations due to HIV/AIDS.

**Results:**

684 patients completed the questionnaire: 231 females and 453 males. The mean age of the sample was 51 years (range 21–78). The mean VAS score was 69.9. 558 patients (81.5%) reported no problems in mobility. 642 patients (93.5%) had no problems in self care. 423 patients (61.8%) had no pain/discomfort while 219 had some problems. 326 patients (46.1%) had some problems in anxiety/depression.

**Conclusions:**

The analysis of self-reported questionnaires indicates that HRQOL in our sample group is not deeply affected by HIV/AIDS. The dimensions that are affected in the least are “mobility” and “self care” while the major problem is “anxiety/depression” with half of the sample reporting moderate or high level.

